# From biomarkers to therapeutic targets: the promise of PD-L1 in thyroid autoimmunity and cancer

**DOI:** 10.7150/thno.50333

**Published:** 2021-01-01

**Authors:** Grégoire D'Andréa, Sandra Lassalle, Nicolas Guevara, Baharia Mograbi, Paul Hofman

**Affiliations:** 1 ENT and Head and Neck surgery department, Institut Universitaire de la Face et du Cou, CHU de Nice, University Hospital, Côte d'Azur University, Nice, France.; 2 Côte d'Azur University, CNRS, INSERM, Institute for Research on Cancer and Aging, FHU OncoAge, Nice, France.; 3 Antoine Lacassagne Cancer Center, FHU OncoAge, Nice, France.; 4 Laboratory of Clinical and Experimental Pathology, Côte d'Azur University and Biobank, Pasteur Hospital, University Côte d'Azur, FHU OncoAge, Nice, France.

**Keywords:** Thyroid autoimmunity, thyroid cancer, PD-1/PD-L1, biomarker, immune checkpoint inhibitors

## Abstract

The programmed cell death-1/programmed cell death ligand-1 (PD-1/PD-L1) immune checkpoint proteins hold promise as diagnostic, prognostic, and therapeutic targets for precision oncology. By restoring antitumor T cell surveillance, the high degree of effectiveness of the immune checkpoint inhibitors (ICIs) has revolutionized cancer treatment. However, the majority of patients (65-80 %) treated with ICIs experience significant side effects, called immune-related adverse events (irAEs), resulting in autoimmune damage to various organs. Therefore, broadening the clinical applicability of these treatments to all cancer types requires an improved understanding of the mechanisms linking cancer immune evasion and autoimmunity. The thyroid is the endocrine gland the most frequently involved in autoimmunity and cancer, the growing incidence of which is raising serious public health issues worldwide. In addition, the risk of developing thyroid cancer is increased in patients with autoimmune thyroid disease and thyroid dysfunction is one of the most common irAEs, especially with PD‑1/PD-L1 blockade. Therefore, we chose the thyroid as a model for the study of the link between autoimmunity, irAEs, and cancer. We provide an update into the current knowledge of the PD‑1/PD-L1 axis and discuss the growing interest of this axis in the diagnosis, prognosis, and management of thyroid diseases within the context of autoimmunity and cancer, while embracing personalized medicine.

## Introduction

**Figure 1:**
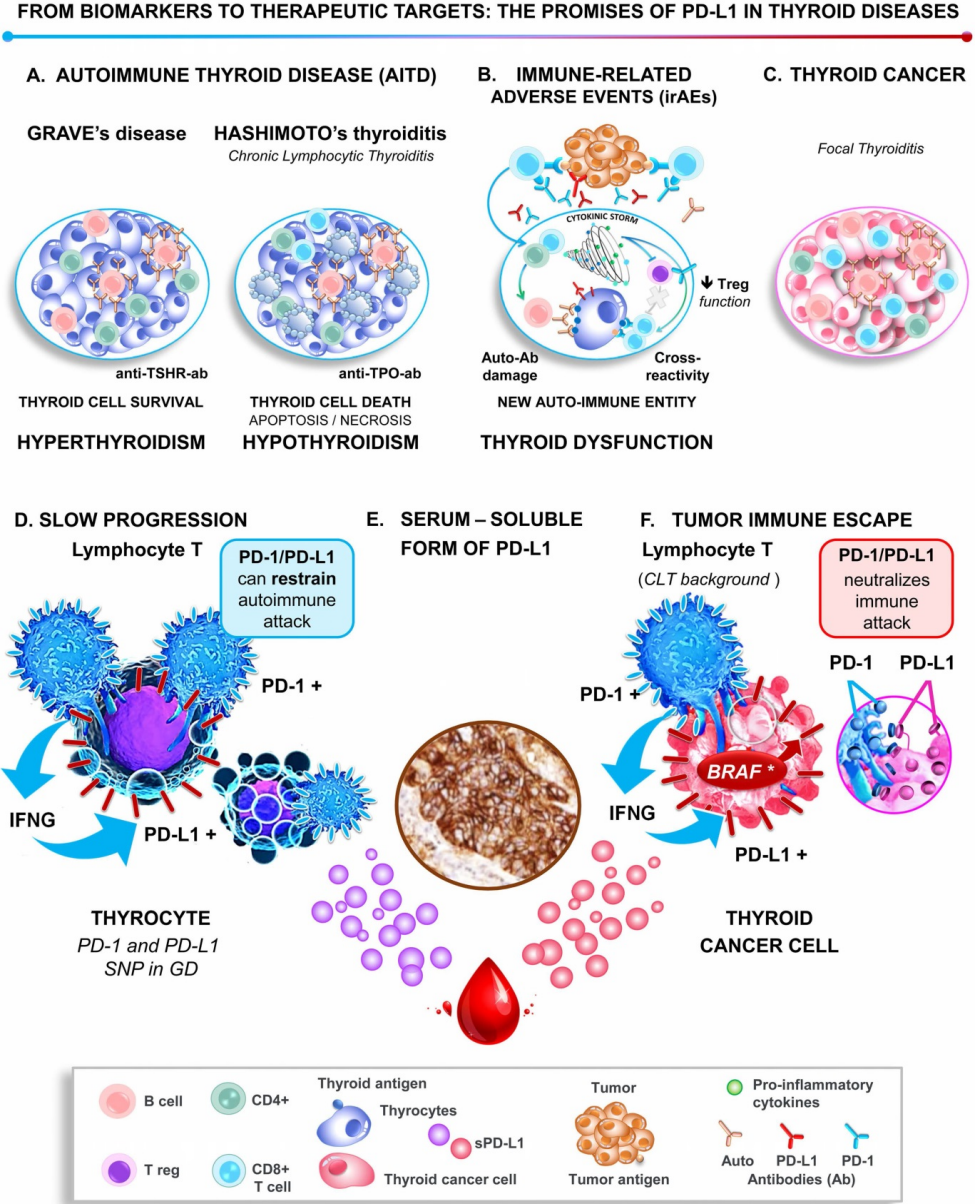
** Role of the PD-1/PD-L1 pathway in thyroid pathogenesis.** *Upper panel* graphical displays the thyroid immune microenvironment in AITD (Graves' disease and Hashimoto's thyroiditis, A), thyroid immune-related adverse events (B), and thyroid cancer (C). *Lower panel*. At the molecular level, the highly activated lymphocytes, which are characterized by the expression of PD-1 and IFNγ, are in close contact with thyrocytes and thyroid tumor cells. Either IFNγ or/and BRAF^V600E^ induce the expression of PD-L1 by the epithelial cells, which binds to its receptor PD-1 on lymphocytes and thereby restrains autoimmune attacks in AITD (D) and promotes thyroid tumor immune tolerance (F). Note that both AITD and TC release the serum-soluble form of PD-L1, which may contribute with the tissue PD-L1 to the immune escape and be considered as biomarkers and therapeutic targets (sPD-L1, E). Abbreviations: AITD, Autoimmune thyroid disease; Anti-TSH-R-ab, Anti-receptor of the thyroid-stimulating hormone antibody; Anti-TPO-ab, Anti-thyroid peroxidase antibody; Auto‑ab, Auto-antibodies; CLT, Chronic lymphocytic thyroiditis; GD, Grave's disease; IFNγ, Interferon-gamma; irAEs, Immune-related adverse events; PD-1, Programmed-cell death receptor 1; PD-L1, Programmed-cell death ligand 1; SNP, Single nucleotide polymorphism.

The thyroid is the endocrine gland the most frequently involved in autoimmunity and cancer 1, 2. The burden due to the rising incidence worldwide of these pathologies represents a major public health issue 1. At a glance, thyroid cancer (TC) and autoimmune thyroid diseases (AITD) appear to involve diametrically opposite immune responses (**Figure 1**). Thyroid cancer progresses, at least in part, because it hijacks the mechanisms of tolerance to immune attack 3. In contrast, AITDs result from the breakdown of the same mechanism of self-tolerance, and the overactive immune response damages the thyroid 4. Of note, the risk of developing thyroid cancer is increased in patients with AITD 4. When immune checkpoint inhibitors (ICIs) are used to treat cancer, the immune system is reinvigorated, resulting in the emergence of significant side-effects called immune-related adverse events (irAEs), which occur frequently in the thyroid gland 5. Therefore, we overview the involvement of the key immune-checkpoint programmed cell death‑1/programmed cell death ligand-1 (PD-1/PD-L1) proteins in cancer and autoimmunity, focusing on the thyroid as a model. We provide an overview of the promise of these proteins in assisting the diagnosis, prognosis, and management of both cancer, and autoimmune diseases of the thyroid, while embracing personalized medicine (**Figure 1**).

A number of breakthroughs have occurred in the last fifteen years that help explain how T cell-mediated immunity is tightly controlled by inhibitory signals, also termed immune checkpoints, which protect healthy tissue from unnecessary and excessive immune damage 6. Multiple immune checkpoints have been identified, including PD-1, which is mainly expressed on activated T cells, but also on regulatory T cells (Tregs), B cells, NK cells, and myeloid cells. Its ligands include PD-L1 and PD-L2. PD-L1 (also called B7-H1 or CD274) is induced at the cell surface of cells of healthy tissues and immune cells in response to various cytokines (notably IFNγ and TNFα), whereas PD-L2 is mainly expressed on activated dendritic cells and macrophages 6, 7. When PD-1 is bound to its ligands PD-L1 and PD-L2, PD-1 switches off T cell antigen receptor (TCR)-signaling, keeping in check the downstream CD8+ T cell survival and cytotoxic attack, while promoting differentiation of CD4+ T-cells into Tregs, to coordinately resolve inflammation 7, 8.

Emerging evidence suggests that the immunosuppressive activity of PD-L1 is not restricted to acute inflammation. Indeed, the PD-1/PD-L1 pathway restrains chronic inflammation and downstream tissue injury in several autoimmune diseases, such as diabetes mellitus type I or systemic lupus erythematosus, among others 9, 10. Similarly, during tumor development, the immune system efficiently recognizes the tumor cells as 'foreign' by detecting the mutated or overexpressed proteins that can produce tumor-specific neoantigens at their cell surface 3, 8. Here again, PD-L1 is expressed by tumor cells to regulate immune attack 7. Herein lies the rationale for blocking checkpoint molecules with immunotherapies (the so-called immune checkpoint inhibitors) to restore antitumor T cell surveillance, now approved by the Federal Drug Administration (FDA) for cancer treatment 7. Nowadays, the major clinical impact of immunotherapies targeting PD-1/PD-L1 has changed the landscape of cancer treatment. However, a substantial percentage of patients do not benefit from these treatments 11. Based on the high response rates of patients with PD-L1-positive tumors, a high level of PD-L1 expression within a tumor was the first FDA approved predictive biomarker for the selection of patients who were likely to respond to anti-PD-1/PD-L1 immunotherapies 12, 13.

## The PD-1/PD-L1 axis in thyroid diseases

Notwithstanding the clinical interest of PD-1/PD-L1 in autoimmunity and malignancies, their role in thyroid diseases remains poorly understood. The purpose of this literature review is to discuss the potential involvement of the PD-1/PD-L1 couple in thyroid pathogenesis and to clarify their role as a biomarker, and therapeutic target, particularly for the challenging management of aggressive thyroid lesions.

### Method of the systematic review

One author (G.D'A) performed a comprehensive literature search of the articles published in English or French using the Medline/PubMed, EMBASE, ResearchGate, and Cochrane Library databases up to May 1, 2020. Manual search through the reference lists of the articles and related reviews was also conducted. The selected studies had to focus on *i*) thyroid dysfunction related to ICIs, *ii*) PD-1/PD-L1 expression in AITD, *iii*) all types of TC, and benign thyroid lesions or *iv*) pre-clinical and clinical use of ICIs for the treatment of TC. Original articles were included, but also the latest reviews and meta-analysis containing the newest or most relevant information. All the methods for measuring PD‑1/PD‑L1 expression on tumor cells and/or tumor-infiltrating lymphocytes (TILs) in primary cancer tissues (on the cell membrane, cytoplasm, or both) were accepted. A total of 162 records were identified, screened, and 106 were excluded (1 Non-English Non-French, 79 irrelevant or not specific enough, 26 duplicate studies). Of the 56 studies included, there were five systematic reviews and meta-analyses. From each included study, the extracted data concerned the characteristics of PD‑1/PD-L1 evaluation, the number of patients and their clinicopathological features and outcome (progression-free survival (PFS) and overall survival (OS)).

### Thyroid immunotoxicity and autoimmunity, the Janus face of anti-PD-1/PD-L1 immunotherapy

The high incidence of autoimmune events (65-79%) in patients treated with ICIs, the so-called immune-related adverse effects, points to safety concerns that cast a cloud over immunotherapy management 14. Autoimmune endocrinopathies are irreversible in 50% of cases, and thyroid dysfunction is the most common irAE, particularly in response to anti‑PD‑1/PD-L1 immunotherapies 14. The spectrum of thyroid dysfunction depends on the type of immunotherapy. In response to anti-PD-1 immunotherapies, they include autoimmune hypothyroidism (2-10% of patients), hyperthyroidism (0.9-7.8%), and thyroiditis (0.34-6.5%), recently acknowledged to be part of the same autoimmune entity 15. Hypothyroidism occurs in 0-10% of patients treated with anti-PD-L1 therapies, while hyperthyroidism (0.5-2%) rarely occurs. As expected, the combination associating anti-PD-L1 and anti-CTLA4, which over activates the immune system, induces a higher prevalence of hypothyroidism (17%) and hyperthyroidism (10%) than mono-immunotherapies 14.

#### Thyroid autoimmune diseases are not a contraindication for immunotherapy

Given the role of PD-1/PD-L1 in limiting autoimmunity 9, 10, 14, patients with preexisting autoimmune Hashimoto's thyroiditis (HT) and/or an asymptomatic elevated titer of thyroid autoantibodies are at-risk of disease worsening or de novo irAEs 16, 17. Paradoxically, the occurrence of irAEs and particularly of thyroid dysfunction emerges as a lesser evil than previously anticipated when treating with ICIs 18.

Indeed, cancer patients with solid tumors who experience irAEs consistently display a significant longer overall survival and overall response rate compared to those lacking toxicity, thus irAE correlates with better prognosis 16, 18-26. Nevertheless, several flaws in the selection of the patients may preclude the establishment of this relationship 27. These include: *i*) as large-sized prospective investigations are missing, the incidence of irAEs varies significantly across studies, as does the tumor type (melanoma being of better prognostic than NSCLC), the tumor staging (over-representation of stages III versus stages IV), and the type of ICI used (anti-CTLA4 being more toxic than anti-PD-1/PD-L1). *Ii*) In addition, patients who live longer ('long-term survivors') are more likely to receive multiple lines of therapy and to develop more irAE than patients who die earlier. However, these confounding factors for recruitment and the latter classical “immortal time bias” can erroneously link better prognosis with the occurrence of irAE. That said, it should be acknowledged that a significant association between irAE and the ICI response persists after adjusting for potential confounders in a multivariable analysis 19, 20. Of interest, regardless of the affected organs (thyroid, skin, lung…), the presence or the treatment of irAEs does not require interruption or compromise ICI treatment 14, 16-18. In light of these observations, it can be assumed that patients with preexisting AITD, and more generally autoimmune or inflammatory diseases, might represent a subgroup of cancer patients who can benefit substantially from anti‑PD‑1/PD-L1 immunotherapies 18. To move forward to personalized management, further large-scale *validation* is needed to determine whether a certain type or severity of an autoimmune or inflammatory disease could point to treatment efficacy and used as a clinical predictive biomarker of anti-PD-1/PD-L1 efficacy.

#### ICI-induced thyroid autoimmunity: a new entity distinct from thyroid autoimmunity

The common clinical presentation and management of irAEs and AITD fuel the hypothesis that they *share an underlying* mechanism (**Figure 1**). Both can be the consequence of the reactivation of the immune system by ICIs by misfiring healthy thyroid cells and killing the 'wanted' tumor cells 14. Downstream of inflammatory attack, the pathogenesis of AITD and irAE involve the secretion of a cytokine storm, that can be integrated into a single cytokine score (the CYTOX), which correlated to immunotherapy toxicity in skin irAE 28. In keeping with this scenario, the most severe forms of both irAEs and AITD are characterized by reduced iodine uptake and increased inflammatory metabolism (as evidenced by diffuse thyroid 18 fluorodeoxyglucose uptake on PET scan image) 17, 29. However, at odds with common pathogenesis, only 22% of patients with thyroid irAEs had elevated thyroid peroxidase antibodies (anti-TPO-ab) compared with >90% reported in Hashimoto's thyroiditis 17, 29. Likewise, the ICI-related thyroiditis resulted from a unique immune phenotype, drastically different from that of HT 16, 17. Indeed, while HT patients exhibited an increase in circulating CD3+, CD4+, and CD8+ T-cell populations, no such increase was observed for ICI-treated patients with irAEs, which instead showed a specific decrease in immature NK cells, and HLA-DR lo/neg immunosuppressive cells 17.

Altogether, the latter differences support the current hypothesis that thyroid irAEs may represent, in part, a new autoimmune entity, even if further studies are needed to improve our knowledge of their specific pathogenesis (**Figure 1**). With the increasing use of cancer immunotherapy, and particularly of the ICI combinations, the incidence and severity of thyroid irAEs are expected to increase, emerging as a significant health concern. Therefore, this as yet unmet clinical need requires non-invasive prognostic approaches that identify patients at risk of thyroid irAEs. As discussed above, some characteristics of routine practice may be helpful in the detection of severe thyroid irAEs, ensuring patient management at an asymptomatic stage 17. Yet, large scale collaborative efforts are critical to robustly define new prognostic biomarkers, as well as to evaluate the occurrence of thyroid irAEs as predictive biomarkers of the response of ICIs.

### PD-1/PD-L1 expression in autoimmune thyroid disease

With an incidence reaching 50/100,000 per year in the female population, autoimmune thyroid diseases are the most prevalent organ-specific autoimmune diseases 2. The two most common AITD are Hashimoto's thyroiditis and Graves' disease 30 that still poses management concerns. In both, an infiltration by immune cells can be observed but is only massive in HT (also known as chronic lymphocytic thyroiditis, CLT) 31. The slow progression of AITD raised questions regarding the interaction of the immune system with thyroid cells 32. Along this line, worsening AITD upon PD‑1/PD‑L1 blockade strongly suggests a critical underlying role for this pathway 9, 14, 33. However, most of the studies into immune checkpoints carried out so far have been conducted in thyroid oncology, and only three studies on this subject in AITD have been published (**Table 1**) 34-37. Lubin *et al.* reported an increased expression of PD-L1 in HT glands, or papillary thyroid carcinoma (PTC) arising in such a background, while they observed little, if any, expression in healthy thyroid tissues 34. It is noteworthy that the immune landscape of thyroid tumors displayed some features similar to AITD and more specifically to CLT. Both humoral- and cell-mediated immune responses are found to be enhanced in the microenvironment of TC and AITD (albeit to a lesser level) compared to benign thyroid nodules 38. Lymphocyte infiltration is observed on histology within and surrounding the tumor, and also throughout the gland. Lymphocytic thyroiditis is characterized by small organized, rather than diffuse, lymphoid follicles, hereafter referred to as focal thyroiditis (FT).

**Figure 2:**
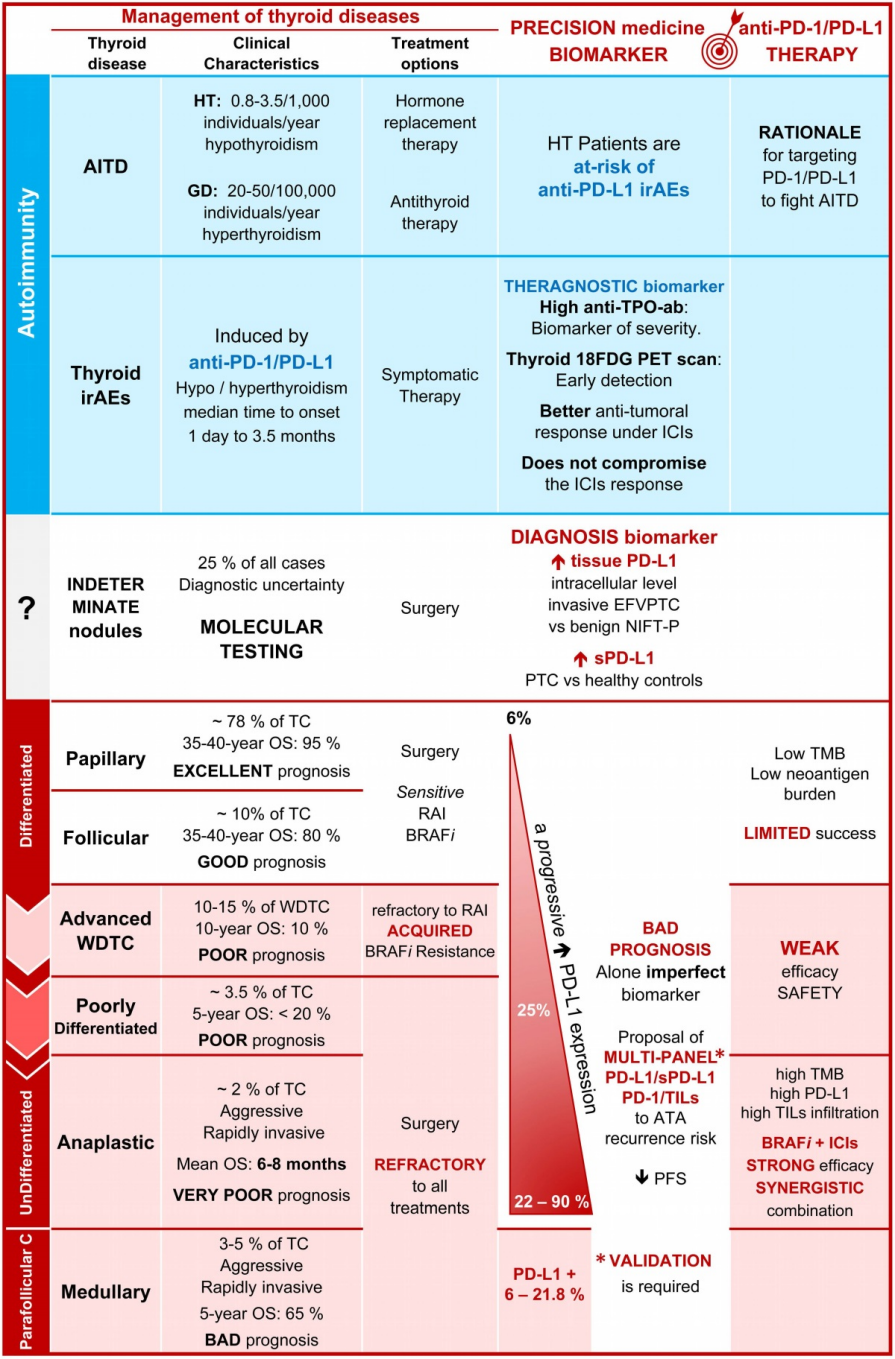
** Promise of PD-L1 in the diagnostic, prognostic, and management of thyroid diseases.** Comparison of some clinical characteristics, and treatment options, of patients with AITD, irAE, and TC emphasizing the percentage of positive cases for PD-L1, the interest of PD-L1 as a diagnosis and prognostic biomarkers as well as the promises of clinical trials using anti-PD‑1 and anti-PD-L1 drugs (*see for details the tables 1-5*). Abbreviations: 18FDG, 18 fluorodeoxyglucose; AITD, Autoimmune thyroid disease; Anti-TSH-R-ab, Anti-receptor of the thyroid-stimulating hormone antibody; Anti-TPO-ab, Anti-thyroid peroxidase antibody; ATA, American thyroid association; BRAFi, BRAF inhibitor; CLT, Chronic lymphocytic thyroiditis; EFVPTC, Encapsulated follicular variant of papillary thyroid carcinoma; GD, Grave's disease; HT, Hashimoto's thyroiditis; ICIs, Immune checkpoint inhibitors; IFNγ, Interferon-gamma; irAEs, Immune-related adverse events; NIFTP, Non-invasive follicular thyroid neoplasm with papillary-like nuclear features; OS, Overall survival; Parafollicular C, Parafollicular carcinoma; PD-1, Programmed-cell death receptor 1; PD-L1, Programmed-cell death ligand 1; PTC, Papillary thyroid carcinoma; RAI, Radioiodine therapy; SNP, Single nucleotide polymorphism; TC, Thyroid carcinoma; TILs, Tumor-infiltrating lymphocytes; PD-1, Programmed-cell death receptor 1; PD-L1, Programmed-cell death ligand 1; PFS, Progression-free survival; SNP, Single-nucleotide polymorphism; TMB, Tumor mutational burden; WDTC, Well-differentiated thyroid carcinoma.

Last year, Álvarez-Sierra *et al.* provided a specific and comprehensive analysis of the PD-1/PD-L1 axis in human AITD 39. They demonstrated using a combined approach that the majority of infiltrating lymphocytes of GD and HT glands were effector and memory CD4+ and CD8+ T cells positive for PD-1. In addition, its ligand, PD-L1 was expressed by the neighboring thyroid follicular cells in 81% of GD glands and 100% of HT glands. Therein the infiltrating T cells produced IFNγ, which was sufficient to induce the expression of PD-L1 *in vitro*. Therefore, as all inflammatory actors were gathered together in the same area, this led to the notion that the PD-1/PD-L1 pathway could be active in AITD glands to safeguard against autoimmune attack. Consistently, polymorphisms in *PD-1* or *PD-L1* were associated with GD (**Figure 1**) 40, 41. The challenge now is to elucidate the cellular mechanisms affecting PD-L1 expression in AITD. Harnessing such an immunosuppressive environment may hold promise as a new therapeutic target to fight autoimmune diseases (**Figure 2**).

### PD-1/PD-L1 expression as a diagnostic biomarker of thyroid cancer over benign lesions

Thyroid carcinomas are the most common cancers of the endocrine gland 1. The incidence has tripled worldwide over the last three decades and is expected to increase further. However, only a small proportion, 5 to 15% are malignant 42. The current diagnostic strategy, mainly built around clinical evaluation, ultrasound evaluation, and fine-needle aspiration cytology (FNAC), can accurately differentiate benign from malignant thyroid nodules in 75% of patients 43, 44. However, a major challenge remains concerning the nodules that fall within the grey-zone of the 'indeterminate' category of the Bethesda classification, which provides the current cytonuclear diagnostic criteria of malignancy 44. In these cases, a repeated FNAC or the use of surgery is recommended, and 60% of nodules are indeed benign as shown on histopathological examination 43, 45. Such diagnostic uncertainty hinders the management of these patients, leading to overtreatment, unnecessary surgery, and an increase in health care costs 46.

#### Diagnostic value of tissue PD-L1

Given the role of PD-L1 in tumor immune escape 47, to date five studies have evaluated its diagnostic value for thyroid nodules, mostly at the protein level (evaluated by immunohistochemistry - IHC) (**Table 2**) 48-52. All these studies showed increased expression of PD-L1 in well-differentiated thyroid carcinomas (WDTC) versus benign lesions (benign nodules, goiter, or follicular adenomas) 34, 48-50, and healthy tissues 34, 49, 50.

Due to its nonspecific cytologic aspects, the encapsulated follicular variant of papillary thyroid carcinoma (EFVPTC) has long been a challenge for diagnosis, as this cancerous lesion was likely to fall into the grey-zone. However, recently, EFVPTC was reclassified into the pre-cancerous 'non-invasive follicular thyroid neoplasm with papillary-like nuclear features' (NIFT-P) 53. Interestingly, PD-L1 expression emerges to be higher in invasive EFVPTC compared to NIFT-P and/or benign nodules 52, 54, in total agreement with their respective reclassification as cancerous and benign lesions 53. Recently, Dell'Aquila *et al.* confirmed the use of PD-L1 as a biomarker of malignancy or aggressive EFVPTC disease, both in tissue biopsies and remarkably in FNAC, compared to NIFT-P 54. Therefore, the authors proposed PD-L1 as a useful biomarker for the diagnosis of NIFT-P and the risk of EFVPTC invasiveness 54. It will be of strong interest to compare the accuracy of PD-L1 to that of ancillary molecular techniques developed for the diagnosis of indeterminate nodules 55, and to determine whether PD-L1 can overcome their complex clinical interpretation 53.

#### Diagnostic value of PD-L1 in serum and FNAC as a non-invasive biomarker

It is worth mentioning that the above PD-L1 IHC analyses of surgery biopsies remain invasive. Thus, exploiting the release by cancer cells into the blood of high amounts of PD-L1 (multiple myeloma, and hepatocellular carcinoma, among others 56, 57, Aghajani *et al.* recently detected a soluble form of PD-L1 (sPD-L1) in the serum (but not in plasma) from PTC patients but not in healthy controls 58.

Altogether, PD-L1 appears to be a helpful diagnostic biomarker for indeterminate nodules, whether at the mRNA or protein level and in tissues or serum 48, 50, 52, 54, 57. Nevertheless, no study has been designed specifically for this subgroup of patients. Therefore, further comparative clinical studies need to be performed with false-positive nodules (benign nodules, or inflammatory/autoimmune thyroid diseases) and TC to ascertain its validity as a specific diagnostic biomarker of malignancy for indeterminate thyroid nodules (**Figure 2**).

### PD-1/PD-L1 expression as a prognostic biomarker in thyroid carcinoma

Approximately 5% of the general population over the age of 60 years' harbors a TC. The majority of TC cases have an excellent prognosis, but a small proportion of patients will experience a more aggressive disease with a risk of recurrence 5, 59. Despite optimal care, distant metastatic spread occurs in 7-23% of cases, and represents the primary cause of thyroid cancer-related deaths with a ten-year OS rate inferior to 50% 5, 59, 60. Among the clinicopathological features, the genomic alterations of *TERT* and *BRAF* have been associated with such a poor TC prognosis, the latter being targeted for the treatment of aggressive TC 5. However, because of limited improvement in patient survival and acquired drug resistance, the usefulness of these genomic biomarkers requires new biomarkers, such as PD-1/PD-L1, which have the advantage of also being targeted by immunotherapies (as discussed later).

#### PD-L1 overexpression supports the progression and recurrence of well-differentiated thyroid carcinoma

Of clinical relevance, the overexpression of PD-L1 appears to predict worse prognosis, identifying patients at-risk of TC progression, representing real hope for the early management of advanced WDTC (18 clinical studies totaling 3107 patients, **Table 3**) 34-37, 48-50, 57, 61-70. Indeed, the expression of PD-L1 independently correlated with all clinicopathological markers of poor TC prognosis 5: such as male gender, an age > 45 years 49, 50, 69, tumor size (including extra-thyroid or multifocal lesions) 36, 49, 57, 63, 66, 69, lymphovascular invasion and lymph node metastasis 34, 36, 49, 62, 64, 65, 69, 70, or TNM stage 48, 50, 63. Not surprisingly given its role in tumor immune escape, PD-L1 overexpression (protein and mRNA) is associated with reduced progression-free survival (PFS) in PTC patients 48, 49, 57, 64, 69. Along this line, its expression tends to persist in up to 50% of the corresponding metastatic lymph nodes, being associated with a higher density of immunosuppressive Tregs, especially in cases of extranodal extension or cancer relapse 50, 64, 65, 71. Importantly, Zhou *et al.* have demonstrated that PD-L1 overexpression could confer a metastatic advantage in WDTC. Silencing *PD-L1* with shRNA delayed follicular thyroid carcinoma (FTC) growth and metastasis in immunocompromised nude mice, which was suggested by the authors to be related to its role in resistance to cell death 63. Of note, independently of its role in immune suppression, PD-L1 was suggested to contribute to glycolysis, stemness, resistance to cell death, and epithelial-to-mesenchymal transition: a pre-requisite for metastasis 72-74. Although critical for therapeutic intervention, the relevance of these potential tumor‑intrinsic PD-L1 effects in human FTC deserves further study 63. Indeed, aside from these encouraging data obtained with mice, we currently lack data evaluating long-term prognosis in patients with lymph node metastasis as a function of a PD-L1 cutoff. Before implementing PD-L1 as a prognostic biomarker and therapeutic target, this critical issue needs to be appreciated to overcome over-diagnosis and treatment of WDTC that would not harm patients.

#### Capitalizing on serum PD-L1 as a robust liquid thyroid cancer prognostic biomarker

Consistent with its prognostic value in other malignancies 56, Aghajani *et al*. showed that a high level of soluble PD-L1 in serum, over 0.44 ng/ml, and its detection in matched tumor tissues were associated with a significantly shorter PFS in PTC patients (uni- and multivariate analyses) 57. As a result, this study places PD-L1 in serum as a marker of bad prognosis for WDTC 57. A longer follow-up time and a larger number of patients are needed to confirm the bad prognostic value of PD-L1 in serum in WDTC.

#### PD-1/PD-L1 expression in aggressive thyroid carcinomas

Aside from the good prognosis of WDTCs, other histological types of TC are of worse prognosis, such as poorly differentiated thyroid carcinomas (PDTC), medullary thyroid carcinomas (MTC), and anaplastic thyroid carcinomas. In agreement with the role of PD-L1 in progression of WDTC, the analysis of nine studies totaling 423 patients (34 PDTC, 85 ATC, and 304 MTC) supports the clinical value of PD-L1 as a biomarker of aggressiveness, and poor prognosis for these three TC subtypes (**Table 4**) 61, 62, 75-81.

*Anaplastic thyroid carcinoma* is a rare malignancy that does not exhibit a long-term response to therapy. Histologically, it is derived from the dedifferentiation of a preexisting PDTC and WDTC. Of interest, a progressive increase in PD‑L1 expression was consistently reported for WDTC (classical and insular PTC variants, PD-L1 positivity in 0-6.1% of positive cases), PDTC (25%) to ATC (22‑90%) 61, 62, 75-81. Mechanistically, the loss of key tumor suppressors (*TP53* and *CDKN2A*) that drive WDTC dedifferentiation correlated with PD-L1 overexpression 82. This is in line with the work of Na *et al.*, who demonstrated that both the expression of immunosuppressive markers (CTLA-4, PD-L1, and HLA-G) and thyroid infiltration by immune cells (ImmuneScore) were upregulated on TC dedifferentiation 75. Given the low number of patients included - which reflects the rarity of these lethal malignancies - very little if any statistical association between PD‑L1 and the other clinicopathological features of bad prognosis, such as the *BRAF^V600E^* mutational status was provided.

Unlike the other differentiated TC, *medullary thyroid carcinoma* is an aggressive cancer derived from parafollicular thyroid calcitonin-producing cells that do not respond to thyroid-stimulating hormone (TSH) suppression or radioactive iodine therapy (RAI), conferring a worse outcome. Of the three studies, PD-L1 positivity was reported in 6.3 to 21.8% of cases and correlated with the other MTC prognostic factors (TNM stage and postoperative calcitonin) 78-80. For a cohort of 201 patients, Shi *et al*. showed that PD-L1 overexpression correlated with recurrent disease (40%), and with a reduced PFS in a multivariate analysis 80. Unfortunately, the two other studies contain major flaws, including the small size of the cohort 78 and a short follow-up period 79, which preclude any statistical correlation of PD-L1 expression with PFS/OS. Similarly to *BRAF*^V600E^, the mutated *RET* protein kinases in MTC cells could regulate the expression of PD-L1 and the subsequent effectiveness of PD-1/PD-L1 blocking therapies, as suggested for melanoma and lung cancers 83, 84. Yet, it is regrettable that none of the studies evaluated the association of PD-L1 with these oncogenic MTC drivers. Here again, the upregulation of the PD-1/PD-L1 pathway in MTC emerged to impact patient prognosis, as it correlated with MTC size, TNM stage, lymph nodes, and distant metastasis 78-80.

However, in agreement with the conclusions of the three previous reviews 69, 85, 86, we acknowledge that alone PD-L1 remains an imperfect biomarker of TC prognosis, correlated 48, 49, 69, 80, 81 or not 36, 61, 62, 77, 78 to PFS/OS. Attention should be paid, however, to the correlation of the expressions of PD-L1/PD-1 on tumor cells and tumor-infiltrating lymphocytes (TILs) with an impaired PFS and OS in patients with ATC 81. Therefore, we would like to nuance this claim by proposing a multi-marker PD-L1 panel by integrating the circulating and tissue levels of PD-L1, with the density of TILs or other clinicopathological features. This multi-marker panel centered around PD-L1 may offer the opportunity to improve the diagnosis of indeterminate lesions, the assessment of early recurrent disease, or the future prediction of treatment (see below) (**Figure 2**).

### Future challenges and uncertainties of PD-L1 as a biomarker of thyroid pathologies

The above compelling studies suggest that the introduction of PD-L1 into clinical practice could improve the diagnosis and the evaluation of the prognosis of TC. However, its real value is not well established, as conflicting results in the percentage of PD-L1 positive tumors have been reported, ranging from 0 51 to 80 % of WDTC 48-50, and up to 90% of ATC 35. To explain these discrepancies, the limitations include:

*i*) The overexpression of PD-L1 alone is not *specific* enough to distinguish the '*true positive*' malignant from *'false-positive'* benign nodules. Indeed, an inflamed microenvironment or an autoimmune pathology (such as HT or GD) can also increase the PD‑L1 expression and lead to misdiagnosis.

*ii*) Across the studies, there is no *standardization* of PD-L1 staining, the primary antibody, and analysis (with a variable *cutoff* and scoring) 87. Among 20 studies, nine different PD-L1 antibodies were used (the commercial E1L3N, E1J2J, Ab174838, MAB1561, MABC290 clones, the complementary SP142 and SP263, and the FDA-approved companion 22C3 antibody) and importantly only 11% of all cases were assessed with FDA-approved PD-L1 diagnostic tests.

*iii*) The interpretation is also complicated by the *cell type* analyzed, as PD-L1 expression was mainly measured on tumor cells, and rarely on tumor-infiltrating immune cells 61, 65, 79.

*iv)* A major technical bottleneck is the *limited sensitivity of anti-PD-L1 towards the mature highly glycosylated* protein 88, leading to '*false negative*' PD-L1 staining at the plasma membrane of thyrocytes 48, 51, but mainly in the intracellular location 35, 48-52, 89, 90. Further harmonization is thus required with new antibodies that recognize the mature PD-L1 cell surface protein.

*v*) Moreover, the spatial heterogeneity and dynamism of PD-L1 expression within an AITD gland or a thyroid cancer make challenging the assessment of its real staining score, in a static picture, in one biopsy, at one specific moment 39. We assume that the longitudinal monitoring of PD-L1 levels in liquid biopsies could help counteract this specific issue 57, 58.

Solving these limitations will be particularly critical when the PD-L1 assay will be systematically tested to optimize the implementation of thyroid precision immunotherapy, as discussed below.

### Clinical efficacy of immune checkpoint inhibitor immunotherapies for thyroid cancer

The PDTC and ATC subtypes, together with refractory WDTC and MTC are devastating diseases that will inevitably progress under standard therapy. In the field of precision thyroid oncology, the *BRAF* oncogene is frequently mutated in TC (25-80%), promoting aggressive tumor growth. Given this tumor *oncogene addiction*, several selective BRAF inhibitors (BRAF*i*, dabrafenib/vemurafenib) were successfully developed. Inevitably, however, the *BRAF*-mutated TCs relapse in only 6-7 months 91, 92. Reactivation of the MEK/MAPK pathway occurs in most drug-resistant patients 92, 93, 94. To bypass this resistance, various combination therapies were developed with MEK (MEK*i*, trametinib) or pan-RAF inhibitors (LY3009120) 92, 93, 94. Of note, the treatment with BRAF*i* and MEK*i* (dabrafenib + trametinib) inhibitors improved patient PFS and OS and was FDA approved for *BRAF*-mutated ATC in 2018 95. However, even with this combination, patients relapse and require new therapeutic options.

The recent advent of PD-1/PD-L1 inhibitors is changing dramatically the prognosis of many refractory cancers that were thought incurable. A high level of infiltration of T lymphocytes (CD8+, CD4+, FoxP3+) within the tumor microenvironment of WDTC positively correlated to PD-L1 expression 35, 49, 50. Similarly, 21.4% of TCs coexist with an autoimmune microenvironment 35, rich in T lymphocytes that can similarly foster WDTC progression through PD-L1 overexpression 34-36, 48, 50. The high level of infiltration of lymphocytes within their microenvironment, together with tumor overexpression of PD-L1, are two characteristics that correlate with the effectiveness of immunotherapy in many solid cancers 61, 62, 76, 81. This provides the molecular basis for immunotherapy for the subset of PTC patients who are refractory to RAI. However, in the beginning, there was some pessimism regarding the utility of immunotherapy in TC management 96, 97. First and foremost, because most thyroid carcinomas are WDTC of good prognosis, and current therapies (mainly surgery and RAI) are effective in controlling the disease. Compared to this strategy, scarce random clinical trials that exploit the PD-1/PD-L1 inhibitors to treat refractory WDTC were met with limited success 98, in line with their low neoantigen burden 99. Likewise, this poor clinical benefit of ICIs should be interpreted with caution as the patients were either heavily pretreated, or not selectively enrolled after the failure of RAI and disease progression.

#### A combination of immune checkpoint inhibitors plus BRAF inhibitors arms the immune system against BRAF^V600E+^ thyroid cancer

ATC is the deadliest TC cancer, and despite aggressive incorporating chemotherapy, radiotherapy, and targeted therapy with BRAF*i*, patients after diagnosis with ATC have a typical survival of only months 91. The recent demonstration that ATC is characterized by a high tumor mutation burden, a high level of PD-L1 expression, a high level of TIL infiltration together with the safety of ICIs 98 all renew the interest in using immunotherapy against this deadly TC 100. However, here again, three prospective clinical trials showed poor response to anti-PD-L1 immunotherapies before rapid progression and death 29, 101-104.

The ongoing challenge is thus to increase the efficacy of ICIs while limiting resistance and adverse effects. Since the initial failure of monotherapies, ICIs are now increasingly tested in combination therapies on patients with aggressive TC. However, while the combinations of ICIs with radiotherapy or chemotherapy are ineffective against ATC 103, 104, it should be pointed out that the *BRAF^V600E^* oncogene controls the overexpression of the PD-1/PD-L1 axis in TC 35, 62, 67, 69, 70, 75, 105. The positive correlation between *BRAF^V600E+^*and PD‑L1/PD-1 expression is of great clinical significance, as it might be used to select TC patients for a *combination of* ICIs with BRAF*i***.** Preliminary results show a superior PFS and OS for the combination of BRAF*i* plus ICIs compared with single-agent treatment. Thirty percent of patients with refractory *BRAF^V600E+^* ATC achieved complete and long-lasting remission with this combination, an impressive result that has never been observed with all current treatments 29, 101, 102. In line with this initial success, evidence from animal models recapitulated the significant tumor shrinkage and a longer OS, with the BRAF*i* + ICIs combination, compared to the lack of efficacy of monotherapies 51, 89, 90.

From a molecular point of view, *how can we explain the robust antitumor effect of the BRAFi + ICI combination*? The biological rationale for the use of BRAF*i* capitalizes on the oncogene addiction of the tumor cells for growth and survival. BRAF*i* targets and kills tumor cells carrying the *BRAF^V600E^* oncogene. Likewise, the expression of the downstream target of the *BRAF* oncogene PD-L1 may decrease at the tumor cell surface. In this case, BRAF*i* therapy would be effective, the tumor would regress, and the combination would not lead to additional antitumor benefit.

However, the targeted BRAF*i* therapy alone is not very effective, and this was associated with the persistence of a high level of tumor PD-L1 expression 66, 106, 107. These intriguing findings generate several hypotheses into the mechanisms of resistance, which, as discussed above, include: *i*) the rapid development of tumor resistance 108, *ii*) polyclonal tumor heterogeneity, or* iii*) poor tumor vascularization 109. All would be associated with intrinsic activation of the MAPK pathway and the downstream expression of PD-L1.

While it is speculative, we suggest that when cancer cells die, they can release tumor antigens within the tumor microenvironment. Once recruited, the 'professional' phagocytes (*i.e.,* dendritic cells and macrophages) ensure the uptake of these tumor-antigens, of the antigen presentation and the priming of T cells 110. Therefore, BRAF*i* can induce '*immunogenic cell death*' that stimulates a cytotoxic immune response against residual tumor cells that survive the initial BRAF*i* treatment 111. Thus, the recent evidence of the immunogenic cell death due to the tyrosine kinase inhibitor (KI) crizotinib is of particular interest 112. In addition to tumor cells, the past decade has provided evidence that BRAF*i* can activate the MAPK pathway of host immune cells that express WT *BRAF*, thereby boosting their activity and tumor infiltration into the tumor. These extrinsic mechanisms fit the reported establishment of an inflamed 'hot' ATC microenvironment with massive T cell infiltration, a high level of production of type I IFN, and indirect tumor upregulation of PD-L1 66, 106, 107.

Collectively, the specific antitumor activity of BRAF*i* combined with its effect on the immune system may support the complex antitumor effects of the targeted therapies and the additive efficacy of BRAF*i* + ICI combination 66, 106, 107. In principle, this synergistic combination would also overcome innate or acquired resistance mechanisms for each drug. In addition to ATC, this combination should therefore be effective against advanced *refractory* WDTC, which shares with ATC the *BRAF^V600E+^* oncogene. In light of these initial positive results obtained with a *limited number of patients*, and the mechanistic recapitulation in a*nimal models*, we strongly encourage further decisive testing of the ICIs + BRAF*i* combination on aggressive CT, including mechanistic studies and prospective randomized clinical trials from multi-centers.

#### Moving forward

More than ever, the field of immunotherapy is experiencing an increase in the use of ICI combinations with other KI such as lenvatinib in the treatment of ATC, which modulate the host immunity, the inflammatory response, and epithelial-to-mesenchymal transition 113. Another combination associates ICIs with RET inhibitors (Nivolumab + Cabozantinib) in treating hepatocellular carcinoma 114, likely by downregulating PD-L1 83. Hu-Lieskovan *et al.* provided a pre-clinical study that helps explain the efficacy of the triple-combination BRAF*i +* MEK*i* + anti-PD-1/PD-L1 in *BRAF^V600E+^*melanoma 115. In line with the effect on the immune system of BRAF*i* in *BRAF^V600E^* thyroid cancers, combining BRAF*i* with MEK*i* shapes the tumor immune landscape by increasing tumor antigen presentation, and by enhancing the migration of effector T cell to the tumor site, which contrasts with single- or dual- combinations. As these infiltrated tumors expressed PD-L1, PD-1 blockade induced *sustained* tumor regression in contrast to mono or dual anti-MAPK targeted therapies 115. Before use in the clinic, the efficacy and side effects of this promising triple-combination BRAF*i +* MEK*i* + anti-PD-1/ PD-L1 are currently being explored in *BRAF* mutated ATC, PDTC or MTC in the ***NCT03181100***clinical trial (**Table 5**). We guess that the ongoing development of combinations of ICIs with inhibitors of oncogenic drivers will provide a major therapeutic breakthrough in the treatment of refractory TC.

#### The hope for future immune checkpoint inhibitor combination strategies

As of May 2020, a survey revealed that sixteen active clinical trials are evaluating immunotherapy targeting the PD-1/PD-L1 axis in unresectable, recurrent and/or metastatic TC after standard care (http://www.clinicaltrial.gov/) (**Table 5**). To unleash massive immune attack within the tumor, ten trials with anti-PD-1, and six with PD-L1 inhibitors, are in the pipeline either as monotherapy or in combination with RAI, KI, radio, and/or chemotherapy, other immunotherapies or even oncolytic bacteria. Through these trials, we hope to be in a unique position to develop an armory of effective anti-PD-1/PD-L1 combinations for managing and defeating deadly TC.

## Summary

Among the endocrine glands, the thyroid is the most frequently involved in autoimmune and cancerous pathologies, two conditions that are increasing worldwide. Compared to previous reviews and meta-analyses focusing on TC 69, 85, 86, we present a comprehensive overview of the shared involvement of the PD-1/PD-L1 pathway of thyroid carcinoma, and also of AITD, and thyroid irAEs (**Figures 1 and 2**). On the one hand, we discussed the implication of the PD-1/PD-L1 brake in restraining the activity of T cells and thereby the progression of AITD in patients who experience a higher risk of developing thyroid irAEs and a better outcome with PD-1/PD-L1 blockade. This may support the biological rationale of acting on immune checkpoints for AITD treatment. Similarly, PD-L1 is hijacked by the cancer cells to evade the immune system.

The *BRAF^V600E^* oncogene, or an inflammatory microenvironment (mostly caused by AITD), can induce PD-L1, which impacts thyroid tumor growth, aggressiveness, and dedifferentiation. As a result, PD-L1 (tissue or liquid) emerges as an attractive *diagnostic*/*prognostic* biomarker. However, alone PD-L1 is not sensitive or specific enough for patient stratification and should be integrated into a multi-marker panel with the other clinicopathological features, to improve the *diagnostic* and *prognostic* accuracy *of TC*, especially in identifying the malignancy of indeterminate nodules. Finally, immunotherapies targeting the PD-1/PD-L1 pathway may represent novel therapeutic promises for advanced, recurrent and/or metastatic thyroid carcinomas, primarily in combination with BRAF*i* (**Figure 2**).

## Conclusion

We have witnessed in the last decade, the revolution of immunotherapy as a real 'life saving' option for cancers that were thought incurable. We guess that the next ten years are going to be as exciting with regard to the translation of the PD-1/PD-L1 pathway from bench to thyroid precision oncology. While much progress has been made, it is fair to say that more advances are needed before the introduction into routine clinical practice of PD-1/PD-L1 as biomarkers and therapeutic strategies. For this purpose, we encourage large scale collaborative efforts to gain further insight into the basic role of PD-L1 in thyroid pathogenesis as well as to overcome the present obstacles of clinically challenging detection, stratification, and resistance to current therapies.

## Figures and Tables

**Table 1:** Frequency of PD-1/PD-L1 IHC expression in autoimmune thyroid diseases and papillary thyroid carcinoma

Histology		PD-L1 positivity			Frequency of PD-1 positivity			References	
Background		Interpretation	Percentage (%)	*p*-value	Percentage (%)	*p*-value			
Benign	No/**CLT**/GD (16/5/16)	**AITD PD-L1** 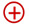	25/**100/81**	*-*	CD4+: 48.6 CD8+: 44.5	0.001	39		
	No/**CLT**/HT (5/5/5)	**PD-L1** **AITD** HT >> CLT 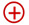	0/**40**/100	< 0.01	*-*	*-*	34		
PTC	No/**FT**/HT (10/10/10)	**PD-L1**, Correlated to HT 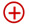	10/**0**/90	0.0001	*-*	*-*			
	**FT Yes**/No (22/99)	**PD-L1**, Correlated to FT 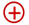	**77.8**/46.5	0.004	100 / 80.8	0.030	35		
		Correlated to TILs	**84.8**/41.9	0.001	100 / 79.6	0.011			
	**FT Yes**/No (33/42)	**PD-L1**, Correlated to FT 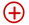	**81.8**/54.8	0.003	*-*	*-*	36		
		Correlated to TILs;T helpers CD4^+^	**78.6**/51.5	0.001	*-*	*-*			
		B cells CD20^+^	**85**/45.7	< 0.001	*-*	*-*			
	FT Yes / No (23 / 58)	**PD-L1** Correlated to FT 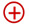	39.1/6.9	0.001	*-*	*-*	37		
									

**Abbreviations**: CLT, Chronic lymphocytic thyroiditis; FT, Focal thyroiditis; GD, Grave's disease; HT, Hashimoto's thyroiditis (defined by authors as specifically as diffuse lymphocytic thyroiditis with follicular cell oxyphilia); IHC, Immunohistochemistry; PD-1, Programmed cell death receptor 1; PD-L1: Programmed cell death-ligand 1; PTC, Papillary thyroid carcinoma; TILs, Tumor-infiltrating lymphocytes. Statistical significance: p < 0.05. A dash is given when the association was not specifically searched for.

**Table 2:** Diagnostic value of PD-L1 expression in thyroid cancer compared to benign lesions

Histology	Total	PD-L1 Staining		Frequency of PD-L1 positivity		References
		Ab (Company)	Interpretation	Percentage (%)	*p*-value	
**Thyroid carcinoma**	185	E1L3N (CST)	**TC >> Benign** Intracellular > Cell surface	66.5 / 40	*-*	48
**Benign nodules**	56			17 / 10		
**Thyroid cancer**, PTC	260	Ab174838 (Abcam)	**WDTC >> healthy** Subcellular localization; Not specified	52.3	*p* < 0.001	49
Corresponding healthy tissue	260	MABC290 (Millipore)		36.9		
**Thyroid cancer**		Ab 82059 (Abcam)	**WDTC > adenomas >> healthy** (quantitative) Intracellular			50
PTC	253			82.5	*IHC qualitative*	
FTC	40			87.5	> 0.05	
**Benign**	119					
Healthy tissue	5			33.3	*IHC quantitative*	
Benign goiters	58			78.4	< 0.0001	
Follicular adenomas	56			84.3	*mRNA* < 0.0001	
***Invasive* EFVPTC**	45	E1L3N (CST)	*Invasive* EFVPTC >> NIFT- P; Intracellular	69	< 0.001	52
**NIFT- P**	52			31		
**Thyroid cancer**	113	22C3 (Dako)	**WDTC > adenomas >> healthy** ***Invasive* EFVPTC>> NIFT- P** Intracellular and cell surface	58.4	*-*	54
PTC	82			59.7		
FTC	2			0		
invasive EFVPTC	29			58.6		
**NIFT- P**	12			0		
**Benign**	81			16		
Goiters	20			0		
Follicular adenomas	48			0		
Oncocytic adenomas	13			100		
**ATC**	20	SP263 (Ventana) / MAB 1561 (R&D)	**↑ ATC progression** > Poorly DC > PTC > healthy (% depends on Ab) Intracellular and cell surface	65 / 90	*-*	51
**Corresponding initial tumor**						
Classical PTC	13			0 / 0		
Poorly differentiated insular tumors	10			0 / 36		
**Corresponding healthy tissue**	17			0 / 23.5		

**Abbreviations**: ATC, Anaplastic thyroid carcinoma; EFVPTC, Encapsulated follicular variant of papillary thyroid carcinoma; FTC, Follicular thyroid carcinoma; IHC, Immunohistochemistry; NIFT-P, Non-invasive follicular thyroid neoplasm with papillary-like nuclear features; PD-L1: Programmed cell death-ligand 1; PTC, Papillary thyroid carcinoma, WDTC, Well-differentiated thyroid carcinoma (including PTC and FTC). Statistical significance: p < 0.05. A dash is given when the association was not specifically searched for.

**Table 3:** Frequency of PD-1/PD-L1 expression in well-differentiated thyroid cancer and their prognostic significance

Histology		PD-1 + /PD -L1 +				Bad prognosis features								Ref	
		Target	Method	Antibody	Frequency (%)	Clinicopathology	FT	T	N	M	Stage	BRAFV600E	Worse PFS		
DTC	92	Tum. PD-L1	IHC	SP142	64	None	-	No	**Yes**	-	-	No	-	68	
			*mRNA*			None	-	No	**Yes**	-	-	No	-		
FTC	40	Tum. PD-L1	IHC	Ab82059	87.5	-	-	-	-	-	-	-	-	50	
	66		IHC	SP142	7.6	-	-	-	-	-	-	-	-	61	
	85		*mRNA*		67.1	None	No	**Yes**	**Yes**	No	**Yes**	-	-	63	
PTC	96	Tum. PD-L1	*mRNA*		42.6	None	No	No	No	No	No	**Yes**	No	62	
	507				-	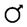	-	**Yes**	**Yes**	No	No	**Yes**	**Yes**	69	
	260	Tum. PD-L1	IHC	Ab174838 MABC290	52.3	Age > 45 y.o. Rich density of TILs 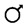	No	**Yes**	**Yes**	-	-	-	**Yes**	49	
	116		IHC	E1L3N	33.6	-	-	-	**Yes**	-	-	-	-	65	
	326		IHC	SP142	6.1	Aggressiveness	No	No	No	No	No	No	No	61	
	165		IHC	E1L3N	66.5	Aggressiveness	**Yes**	-	-	-	Yes*	-	**Yes**	48	
	253		IHC	Ab82059	82.5	Age > 45 y.o. Rich density of TILs	**Yes**	No	No	No	No	-	-	50	
			*mRNA*				-	No	No	No	**Yes**	-	-		
	75		IHC	22C3	66.7	Lymphovascular invasion	**Yes**	**Yes**	No	-	No	-	No**	36	
	81		IHC	SP263	16.4	None	**Yes**	No	No	No	No	-	-	37	
	30		IHC	E1J2J	33	None	**Yes**	-	**Yes**	-	-	-	-	34	
	33		IHC	4059	-	-	-	-	-	-	-	**Yes**	-	67	
	126	Tum. PD-L1	IHC	SP142	53.2	Rich density of TILs No psammoma bodies No stromal calcification	**Yes**	No	No	-	-	Yes***	-	35	

		***TIL PD-1***		UMAB199	84.9		***Yes***	No	No	-	-	No	-		
	110	Tum. PD-L1	IHC	SP142	46	No psammoma bodies	-	No	No	-	No	**Yes**	-	66	
		***TIL PD-1***		UMAB199	78	No stromal calcification	-	***Yes***	No	-	No	***Yes***	-		
	25	Node PD-L1	*Flow cytometry*		-	-	-	-	**Yes**	-	-	-	**Yes**	64	
	101	Serum sPD-L1	*ELISA*		-	None	No	**Yes**	No	-	No	-	**Yes**	57	

**Abbreviations**: DTC, Differentiated thyroid carcinoma; ELISA, Enzyme-linked immunosorbent assay; FT, Focal thyroiditis; FTC, Follicular thyroid carcinoma; IHC, Immunohistochemistry; M, Metastasis; N, Nodes; PD-1, Programmed cell death receptor 1; PD-L1: Programmed cell death-ligand 1; PFS, Progression-free survival; PTC, Papillary thyroid carcinoma; T, Tumor; TILs, Tumor-infiltrating lymphocytes; Tum., Tumoral expression. 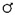 Male gender. Statistical significance: p < 0.05. A dash is given when the association was not specifically searched for. * When stage I is compared to stages > I, ** Median PFS was significantly lower in the PD-L1+/CD8+low subgroup, *** After excluding PTC cases with a background of CLT.

**Table 4:** Frequency of PD-1/PD-L1 expression in aggressive thyroid carcinomas and their prognostic significance

Histology		PD-1+ / PD-L1+				Correlation with bad prognostic features								Ref
		Target	Method	Antibody	Frequency (%)	Clinicopathology	T	N	M	Stage	BRAFV600E	Worse PFS	OS	
PDTC	6	Tum. PD-L1	IHC	SP142	0	Histology	No	No	No	No	-	No	-	61
	28		IHC	E1L3N	25	None	**Yes**	-	-	-	-	No	No	77
ATC	11	Tum. PD-L1	*mRNA*		27.3	-	-	-	-	-	-	-	-	62
	9		IHC	SP142	22.2	Histology	No	No	No	No	-	No	-	61
	49		IHC	E1L3N	28.6	-	-	-	-	-	No	-	-	76
	16	Tum. PD-L1	IHC	E1L3N	81.3	-	-	-	-	No	-	*Trend*	*Trend*	81
		**TILs PD-1**	IHC	NAT105	100	-	-	-	-	No	-	No	**Yes**	
MTC	16	Tum. PD-L1	IHC	SP263	6.3	None	No	No	-	No	-	No	No	78
	87	Tum. PD-L1	IHC	SP263	21.8	None	No	No	**Yes**	No	-	-	-	79
		TIL PD-1	IHC	MRQ-22	25.3	None	No	No	No	No	-	-	-	
		**PD-1+/PD-L1+**				None	No	No	**Yes**	**Yes**	-	-	-	
	201	Tum. PD-L1	IHC	22C3	14.4	None	**Yes**	**Yes**	-	**Yes**	-	**Yes**	-	80

**Abbreviations**: ATC, Anaplastic thyroid carcinoma; IHC, Immunohistochemistry; M, Metastasis; MTC, Medullary thyroid carcinoma; N, Nodes; OS, Overall survival; PD-1, Programmed cell death receptor 1; PD-L1: Programmed cell death-ligand 1; PDTC, Poorly differentiated thyroid carcinoma; PFS, Progression-free survival; PTC, Papillary thyroid carcinoma; T, Tumor; TILs, Tumor-infiltrating lymphocytes; Tum., Tumoral expression. PD-1+/PD-L1+ means that the co-expression of PD-1 and its ligand has been taken into account 79. Statistical significance: *p* < 0.05. A dash is given when the association was not specifically searched for.

**Table 5:** Ongoing worldwide clinical trials evaluating anti-PD-1/PD-L1 immunotherapies against advanced thyroid cancers

ICIs		Combination	Condition	Country	Estimated enrollment	Study Phase	NTC Number
Anti-PD-1	Nivolumab	± Anti-CTLA-4	Rare tumors	USA	818	2	NCT02834013
		(Ipilimumab ®)	*RAI refractory TC*	USA	54	2	**NCT03246958**
	Pembrolizumab	-	Advanced refractory solid tumors	Worldwide	1395	2	NCT02628067
			Advanced refractory rare solid tumors	France	350	2	NCT03012620
			*ATC*	USA	20	2	**NCT02688608**
			***Advanced unresectable MTC***	USA	30	2	**NCT03072160**
		***± Chemo-Radiation ± Surgery*** ***(Docetaxel + Doxorubicin + IMRT)***	***ATC***	USA	NA	2	**NCT03211117**
		± Chemotherapy (Docetaxel)	Poorly chemo-responsive TC and salivary gland cancer	USA	46	2	NCT03360890
		***± RTKi* (Lenvatinib ®)**	***Advanced refractory DTC***	USA	60	2	**NCT02973997**
		± Oncolytic bacteria	Advanced refractory solid tumors	USA	18	1	NCT03435952
		Clostridium Novyi-NT					
Anti-PD-L1	Atezolizumab	***± BRAFi (Vemurafenib ®)***	***PDTC - ATC - MTC***	USA	50	2	**NCT03181100**
		***± MEKi (Cobimetinib ®)***					
		***± VEGFi (Bevacizumab ®)***					
		***± Chemotherapy (Nab-paclitaxel/Paclitaxel)***					
		± RTKi (Cabozantinib ®)	Advanced solid tumors	Europe, USA	1732	1/2	NCT03170960
	Avelumab	± Anti-OX40 (PF-0451860) ± Anti-TNFRSF9 (Utomilumab ®) ± Radiotherapy	Advanced refractory solid tumors	USA	184	1/2	NCT03217747
	Durvalumab	***± RAI***	***Advanced DTC***	USA	NA	1	**NCT03215095**
		***± Anti-CTLA-4 Tremelimumab***	***Advanced refractory DTC***	Spain	46	2	**NCT03753919**
		***± Anti-CTLA-4 Tremelimumab*** ***± Radiotherapy***	***Metastatic ATC***	USA	NA	1	**NCT03122496**

**Abbreviations**: ATC, Anaplastic thyroid carcinoma; BRAFi, BRAF inhibitor; DTC, Differentiated thyroid carcinoma; IMRT, Intensity-modulated radiation therapy; MEKi, MEK inhibitor; MTC, Medullary thyroid carcinoma; NA, Non specified data; PD-1, Programmed cell death receptor 1; PD-L1: Programmed cell death-ligand 1; PDTC, Poorly differentiated thyroid carcinoma; PTC, Papillary thyroid carcinoma; RAI, Radioiodine therapy; RTKi, Receptor of tyrosine kinase inhibitor; TC, Thyroid carcinoma; VEGF*i*, VEGF inhibitor. Studies in bold and italics were designed specifically for TC, while the others included all subtypes of TC.

## Abbreviations

18FDG: 18-Fluoro-deoxyglucose
AITD: Autoimmune thyroid diseases
Anti-TPO-ab: Anti-thyroid peroxidase antibodies
Anti-TSH-R-ab: Anti-thyrotropin receptor antibodies
ATC: Anaplastic thyroid carcinoma
BRAF*i*: BRAF inhibitors
CLT: Chronic lymphocytic thyroiditis
EFVPTC: Encapsulated follicular variant of papillary thyroid carcinoma
FDA: Federal drug administration
FTC: Follicular thyroid carcinoma
GD: Graves' disease
HT: Hashimoto's thyroiditis
ICIs: Immune checkpoint inhibitors
IHC: Immunohistochemistry
irAEs: Immune-related adverse events
KI: kinase inhibitors
MTC: Medullary thyroid carcinoma
NIFT-P: Non-invasive follicular thyroid neoplasm with papillary-like nuclear features
OS: Overall survival
PD-1: Programmed cell death receptor 1
PD-L1: Programmed cell death-ligand 1/B7-H1/CD274
PDTC: Poorly differentiated thyroid carcinoma
PFS: Progression-free survival
PTC: Papillary thyroid carcinoma
RAI: Radioactive iodine therapy
sPD-L1: Soluble form of PD-L1
TC: Thyroid cancer/carcinoma
TCR: T cell antigen receptor
TILs: Tumor-infiltrating lymphocytes
Tregs: Regulatory T cells
WDTC: Well-differentiated thyroid carcinoma
